# Prediction of glaucoma severity using parameters from the electroretinogram

**DOI:** 10.1038/s41598-021-03421-6

**Published:** 2021-12-13

**Authors:** Marc Sarossy, Jonathan Crowston, Dinesh Kumar, Anne Weymouth, Zhichao Wu

**Affiliations:** 1 Ophthalmology, Department of Surgery, University of Melbourne, Melbourne, VIC Australia; 2 Duke-NUS Medical School, Singapore, Singapore; 3 RMIT University, Melbourne, Australia; 4 Department of Optometry and Vision Sciences, The University of Melbourne, Melbourne, Australia; 5 Centre for Eye Research Australia, Royal Victorian Eye and Ear Hospital, East Melbourne, Australia

**Keywords:** Glaucoma, Neurophysiology

## Abstract

Glaucoma is an optic neuropathy that results in the progressive loss of retinal ganglion cells (RGCs), which are known to exhibit functional changes prior to cell loss. The electroretinogram (ERG) is a method that enables an objective assessment of retinal function, and the photopic negative response (PhNR) has conventionally been used to provide a measure of RGC function. This study sought to examine if additional parameters from the ERG (amplitudes of the a-, b-, i-wave, as well the trough between the b- and i-wave), a multivariate adaptive regression splines (MARS; a non-linear) model and achromatic stimuli could better predict glaucoma severity in 103 eyes of 55 individuals with glaucoma. Glaucoma severity was determined using standard automated perimetry and optical coherence tomography imaging. ERGs targeting the PhNR were recorded with a chromatic (red-on-blue) and achromatic (white-on-white) stimulus with the same luminance. Linear and MARS models were fitted to predict glaucoma severity using the PhNR only or all ERG markers, derived from chromatic and achromatic stimuli. Use of all ERG markers predicted glaucoma severity significantly better than the PhNR alone (*P* ≤ 0.02), and the MARS performed better than linear models when using all markers (*P* = 0.01), but there was no significant difference between the achromatic and chromatic stimulus models. This study shows that there is more information present in the photopic ERG beyond the conventional PhNR measure in characterizing RGC function.

## Introduction

Glaucoma is a major cause of irreversible vision loss throughout the world. It is a neuropathy of the optic nerve^1^ with progressive loss of fibres of the nerve and cell bodies of the retinal ganglion cells (RGCs). Intraocular pressure (IOP) remains the only modifiable risk factor in glaucoma, and the reduction of IOP via topical medical therapy or surgical intervention remains the main approaches for slowing the progression of the disease. Advanced glaucoma has characteristic changes of visual field and optic nerve structure, but the early changes with glaucoma can be difficult to detect. It has been estimated that at least 25 to 33% of RGCs must be lost before producing significant visual field abnormalities^2^.

Prior to ganglion cell death, RGCs have been shown to exhibit structural and functional changes^3^. Optic nerve crush^4^ and acute IOP elevation^5^ studies in the mouse showed structural changes that include a reduction in the dendritic arbor area, the length of dendrites and the number of dendrites, which are correlated with the severity of the disease. Early functional changes that occur in sick RGCs include an increased excitability^6^, which can manifest as an increased basal or stimulated firing rate^7^. This may be caused by a depolarization of the resting membrane potential reducing the threshold for excitation^8^. In primate eyes with experimental glaucoma, the RGCs become less responsive with the mean and peak spike rates falling^9^. With continued stress, the ultimate outcome for the stressed RGCs is apoptosis^10^. Capturing early functional changes of the RGCs could thus aid in predicting glaucoma progression and provide complementary information in the early diagnosis of this condition.

The electroretinogram (ERG)^11^ is an electrical response measured at the cornea from a photic stimulus of the eye, and it is a direct measure of retinal function that could be used to capture early RGC dysfunction. Techniques described to measure such function include the pattern ERG (PERG), the positive scotopic threshold response (pSTR) and the Photopic Negative Response (PhNR)^12^. By following a cohort of people considered glaucoma suspects, Banitt^13^ showed that changes in the PERG preceded structural changes as measured on the optical coherence tomography (OCT) by a number of years. Working in a rat model, Liu^14^ showed that with chronic IOP elevation, pSTR dropped by 25% with no significant change in RGC density.

The photopic negative response (PhNR) of the ERG is a slow negative potential following the b-wave. First described by Viswanathan et al.^15^ in 1999, it has been shown to be reduced in glaucoma, both in clinical studies and in animal models^16–20^ and Ref.^21^. It arises from the spiking potentials of retinal ganglion cells. It is elicited under photopic conditions by a red flash of light on a blue background and recorded from a corneal electrode relative to the lateral canthus of the eye. Previous studies have shown that there are significant correlations between the PhNR amplitude and the mean deviation (MD) of standard automated perimetry (SAP)^22^ and peripapillary nerve fiber layer thickness^23^. The PhNR may thus be a useful measure for the objective assessment of RGC function in glaucoma.

Of note, the slow negative potential after the b-wave (the PhNR) is interrupted by a positive peak termed the i-wave of the ERG^24^. This waveform has a variable size and is poorly understood, but it is conserved in primate and non-primate species and thought to arise from cells distal to the RGCs and from the off pathway^25^. The presence of the i-wave can lead to different interpretations of the PhNR wave and therefore can affect the measurement of PhNR amplitude and latency. Recently, the PhNR has been subdivided into: (i) PhNR1, the trough after the b-wave and before the i-wave, and (ii) PhNR2, the first trough after the i-wave^26^ (with the latter being typically measured as the PhNR). There has been little published on the significance of the i-wave itself, although authors have noted that it increases in prominence or amplitude in the presence of RGC pathology^25^. Capturing these additional parameters of the ERG beyond the conventional PhNR measure could also help better capture RGC dysfunction.

In addition, the stimulus parameters for optimizing the PhNR for the characterization of RGC dysfunction have been studied. Sustar^27^ showed that the PhNR elicited with a chromatic stimulus was better at discriminating between glaucomatous and non-glaucomatous eyes than an achromatic stimulus. Hara^28^ reported that the correlations between the PhNR1 and the mean deviation were stronger for the achromatic stimulus but the converse was true for the PhNR2, where the correlations were stronger for the achromatic stimulus. It is likely that these observations relate to the changes in the i-wave between the ERGs elicited by chromatic and achromatic stimuli, which they reported as occurring earlier later in the achromatic compared to chromatic stimulus, and thus had less of an impact on the PhNR I amplitude. Nonetheless, they reported that the PhNR II of the chromatic stimulus performed the best at discriminating between eyes with and without glaucoma. However, neither of these two studies have compared the utility of these three parameters—the PhNR1, i-wave and PhNR2—when used in combination (rather than in isolation) between the chromatic and achromatic stimuli. It is plausible that the utility of the achromatic stimulus may improve when these three parameters are considered in combination, due to its ability to more clearly demarcate the PhNR1 and i-wave.

We thus sought to examine whether the use of a fuller set of parameters (a-wave, b-wave, i-wave, PhNR1 and PhNR2) elicited using both chromatic and achromatic stimuli could better capture RGC functional loss compared with the use of the PhNR alone. This was performed by evaluating its predictive performance for a combined structural and functional measure of disease severity in glaucoma by Meideiros et al.^29^, termed the estimated RGC (eRGC) count. Incorporating all markers into a predictive model allows for normalization of overall gain via the a- and b-wave amplitudes and the effect of the i-wave on the estimation of the PhNR via the use of the i-wave itself and the PhNR1 and PhNR2. In this study, we examined both a conventional linear predictive model and a Multivariate Adaptive Regression Splines (MARS) model. The MARS approach models the outcome variable as a linear sum of piecewise linear functions (truncated functions with knots)^30^. As such, it can be used to model the outcome measure as a linear sum of functions that can saturate or change their significance depending on amplitude, and allow intuition about the underlying process without incorporating interaction terms. We thus sought to use MARS as it could better model potentially complex functions for each of the ERG parameters to achieve improved prediction performance and model intuition.

## Methods

The study was an approved study by the Human Research Ethics Committee of the Royal Victorian Eye and Ear Hospital, and it was conducted in accordance with the Declaration of Helsinki. All participants provided written informed consent prior to any study procedures being undertaken.

### Participants

Participants with primary open angle glaucoma were recruited from a private ophthalmology practice, and the diagnosis of glaucoma was based on a comprehensive clinical assessment by an ophthalmologist based on characteristic optic nerve head appearance, the presence of glaucomatous visual field defects and/or neuroretinal tissue loss on OCT imaging. No participants with ocular or systemic diseases that could affect the optic nerve such (as choroidal neovascular membrane, extensive macular atrophy, diabetic retinopathy, multiple sclerosis or epiretinal membrane) were included and only adult patients over the age of 18 were eligible for inclusion in this study. Both eyes were included where glaucoma was bilateral, and only eyes with an acuity of 20/40 (LogMAR 0.3) or better were included in the study.

### Standard automated perimetry

All participants performed standard automated perimetry (SAP) testing using the 24-2 Swedish Interactive Threshold Algorithm (SITA) Fast protocol on the Humphrey Field Analzyer 3 (Carl Zeiss Meditec Inc.; Dublin, CA), following correction of the spherical refractive error component from subjective refraction. All visual field results had fixation losses and false negative responses of ≤ 33% and false positives of ≤ 20%.

### Optical coherence tomography imaging

All participants also underwent optic disc-centered OCT volume scans performed with the Cirrus HD-OCT device (Carl Zeiss Meditec Inc.; Dublin, CA) with dilated pupils. This model has superluminescent diode illumination and obtains 27,000 A-scans per second, with an overall axial resolution of 5 µm. A scab centred on the optic disc was obtained consisting of 200 × 200 A-scans and covering an area of 6 × 6 mm. The circumpapillary retinal nerve fiber layer (RNFL) thickness was derived from this cube from a derived 3.46 mm diameter circle scan, consisting of 256 A scans with segmentation performed by the instrument. All scans had a signal strength score ≥ 7, and only those showing correct centration and accurate segmentation, were analysed in this study.

### Estimated retinal ganglion cell counts

For each eye, an estimate was made of the RGC counts (eRGC) as described by Medeiros et al.^29^. This model is based on empirical formulas developed by Harwerth et al.^31^. From non-human primates, Harwerth developed a linear model estimating the RCG soma density (somas/mm^2^) loss in dB units at location k ($g{l}_{k}$) at an eccentricity ($ec$) from the SAP visual sensitivity ($s$; dB) via slope ($m$) and intercept ($b$).

1 $${m}_{k}=0.054e{c}_{k}+0.91,$$

2 $${b}_{k}=-1.5e{c}_{k}-14.8,$$

3 $$g{l}_{k}=\left({s}_{k}-{b}_{k}\right)/{m}_{k}.$$

Translating the non-human primate work to humans required accounting for the different length of the eye in humans vs. non-human primates and the change in visual field threshold strategy from full threshold to SITA. For humans using SITA, the equations became

4 $${\mathrm{m}}_{\mathrm{k}}=0.07128e{c}_{k} + 0.91,$$

5 $${b}_{k}=-1.98e{c}_{k}-14.8,$$

6 $$g{l}_{k}=\frac{{s}_{k}-1-{b}_{k}}{{m}_{k}}+4.7.$$

The total ganglion cell number SAPrgc is found as

7 $$\mathrm{SAPrgc}={\sum }_{k=1}^{K}{10}^{0.1g{l}_{k}}.$$

Noting that the number of ganglion cell axons entering the optic nerve from an area of the retina must be representative of the number of cell bodies, the authors also developed an estimate of axon numbers from the circumpapillary measurements of RNFL thickness measured by OCT. Recognizing a near linear loss of axons with normal aging and remodelling of the nerve fiber layer, the authors fitted the following formula relating axon density d (axons/μm^2^) for a patient of age (ag) in years for a section of the RNFL scan of length px in pixels and average height (mh; μm). A 21.2 pixel length per pixel over a 10.87 mm scan length was used.

8 $$d=-0.007ag+1.4.$$

And a total axon count a given simply as

9 $$a=21.2 mh\, px\, d.$$

Again, to account for the effect of remodelling in glaucoma and to express the final estimate in terms of total ganglion cell axons from OCT imaging (OCTrgc), a mean deviation correction *c* is calculated from the MD in dB as

10 $$\mathrm{c}=-0.26\, \mathrm{MD} +0.12.$$

And using the *d* from Eq. (8), the OCT model is

11 $$\mathrm{OCTrgc}={10}^{0.1\left[10lo{g}_{10}\left(10870 d rnfl\right)-c\right]},$$

where *rnfl* is the mean RNFL thickness in μm from the OCT.

Medeiros et al.^29^ finally proposed the use of a weighted mean reflecting the inverse relationship between disease severity of SAP and OCT estimates where the MD ranged from 0 to − 30 dB to obtain a final estimated RGC count in an eye eRGC as

12 $$\mathrm{eRGC}=\left(1+\frac{MD}{30}\right)\mathrm{OC}{T}_{rgc}-\frac{MD}{30}\mathrm{SA}{P}_{rgc}.$$

Equation (12) was used for the RGC count estimate in this study.

### Electroretinography

Both eyes were dilated with 1% tropicamide drops prior to recording. Three strand Dawson, Trick, and Litzkow (DTL)^32^ wire electrodes were placed in the lower conjunctival fornices. Local anaesthetic drops were placed prior (1% tetracaine). Gold cup skin electrodes (Grass) were used for ground and indifferent electrodes. The indifferent electrodes were placed at the lateral canthi and the ground electrode was placed on the forehead. Impedances of all electrodes including the ground were checked using the inbuilt meter and were accepted if the impedance was less than 5 kΩ.

An Espion E3 system with the ColorDome ganzfeld stimulator (Diagnosys LLC; Lowell, MA) was used to collect the ERG data. For the first step, a blue background of 10 cd/m^2^ (peak wavelength 465 nm) was used for a preadaptation time of 2 min. A total of 125 flashes were then presented at a rate of 2 Hz, with a luminance of 1 cd s/m^2^ using a red flash (peak wavelength 635 nm) of 4 ms duration. This step was compliant with the International Society for the Clinical Electrophysiology (ISCEV) extended protocol for the PhNR^33^. Step 2 used the same photopic intensity of flash and background, but it was delivered as a white-on-white stimulus after a further 2 min preadaptation to the achromatic background. The stimuli were also presented at a rate of 2 Hz, with a 4 ms duration. The signals were collected with a sample rate of 4 kHz (which is higher than the minimum 1 kHz sample rate specified by the ISCEV protocol for recording the ERG) and an epoch length of 150 ms, and with 30 ms of pre-stimulus recording. Both raw data and filtered (band pass 0.3–100 Hz) were recorded and signals with an amplitude of over 150 µV were rejected.

### Signal processing

**Figure 1:**
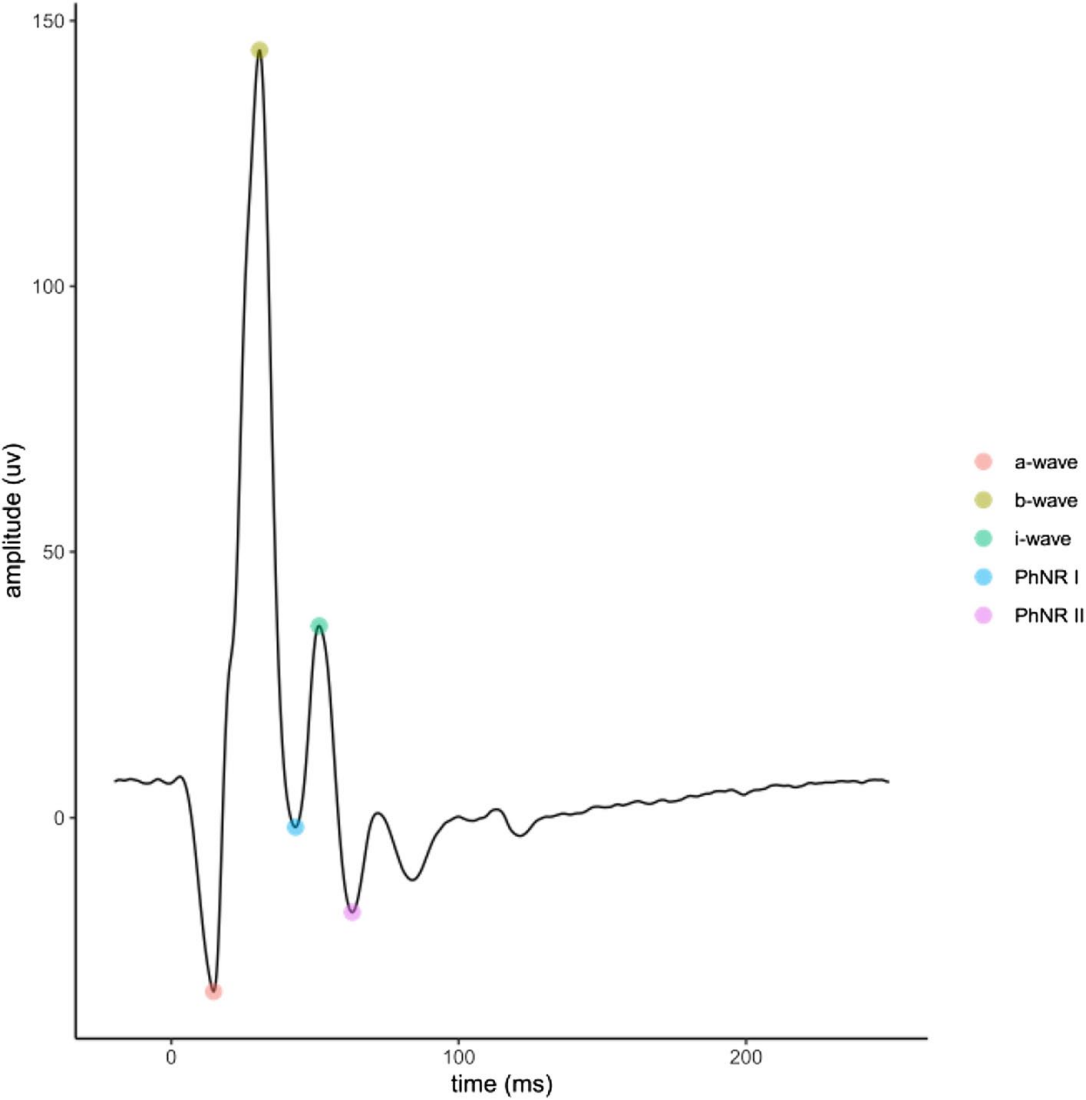
Typical electroretinogram trace (ERG) for the first step of red flash on blue background. Automatic marker placement is shown.

The location of the a-wave and b-wave from the signal were determined by locating the b-wave as the global maximum and then the a-wave as the minimum in the segment between the start of the vector and the b-wave. The i-wave was located by determining the first peak after the b-wave. The PhNR1 and PhNR2 were determined to be the negative going troughs either side of the i-wave. A typical ERG averaged trace elicited from chromatic stimuli is shown in Fig. 1.

### Statistical analysis

The Caret^34^ package in R was used for predictive model fitting and tuning. Linear and multivariate adaptive regression splines (MARS) were fitted. Overfitting was avoided by using tenfold cross-validation. For the MARS models, the degree was set to 1 and the maximum number of terms was set to 40. The degree is the level of interaction of terms allowed—in this case none allowed. An example second degree interaction term would be the a-wave amplitude multiplied by the b-wave amplitude. The number of terms in the model is a function of the number of input features and the number of breaks in the hinge function. Without a breakpoint, a single feature will have 2 terms: intercept and gradient. With a single breakpoint, this becomes 4 terms: intercept, the 2 gradients and the breakpoint location.

For each of the tuned ‘final models’, the prediction performance of the ERG for the eRGC counts was examined based on the proportion of variance explained (R^2^) by the model. Each model was compared to the base case of the PhNR only model. The significance of difference was determined using a bootstrap technique (*n* = 1000 resamples) which dealt with the within-subject correlations through resampling at the individual level.

## Results

A total of 103 eyes with glaucoma from 55 participants were included in this study, and their characteristics are shown in Table 1.

**Table 1:** Characteristics of the individuals and eyes with glaucoma in this study.

Characteristics	(103 eyes from 55 individuals)
**Individual level**	
Age (years)	75 (66 to 80)
Gender (female)	21 (38%)
Diabetes (present)	7 (13%)
Hypertension (present)	35 (64%)
**Eye level**	
Refraction sphere (D)	0.00 (− 1.00 to 0.50)
Visual acuity (logMAR)	0.0 (− 0.1 to 0.1)
Intraocular pressure (mmHg)	15.0 (12.0 to 16.0)
Mean deviation (dB)	− 2.5 (− 5.9 to − 0.5)
Retinal nerve fiber layer thickness (µm)	73.0 (62.8 to 82.5)
eRGC (‘000s)	601 (470 to 753)

Continuous statistics presented as median and interquartile range, categorical statistics as number and percentage.

*logMAR* logarithm of the minimum angle of resolution, *eRGC* estimated retinal ganglion cell count.

### Prediction of estimated retinal ganglion cell counts

The PhNR/B ratio gave poor predictive performance with R^2^ values ranging from 0.028 for the linear chromatic model through to 0.073 for the MARS chromatic model. Table 2 summarizes the predictive ability of the different models using the ERG for the eRGC measure for the PhNR alone and the full set of markers. The predictions of eRGC were significantly better with the models utilising the full feature set from the ERG, compared with those using the PhNR alone (*P* ≥ 0.02 for all). The predictive performance of the MARS models was also significantly better than the linear models using the full set of markers (*P* = 0.01 for both), but not when using the PhNR alone (*P* ≥ 0.14 for both). Finally, the models derived from ERG recordings with achromatic stimuli performed better at predicting eRGC counts than ones using chromatic stimuli, but it this difference did not reach statistical significance (*P* ≥ 0.19 for all).

**Table 2:** Model performance as proportion of variance explained (R^2^) for the prediction of ganglion cell count from models based on photopic negative response (PhNR) alone or the full set of amplitude features (“Markers”, including the a-, b- and i-waves and PhNR1 and PhNR2). Both simple linear regression and multivariate adaptive regression spline (MARS) models are shown. Testing was with red flashes on blue background (chromatic) or white on white (achromatic). P-values were calculated by bootstrap resampling.

	*R*^2^		*P*-value for comparison between				
			PhNR vs. markers		Linear vs. MARS	Chromatic vs. achromatic	
	Linear	MARS	Linear	MARS		Linear	MARS
**Chromatic**							
PhNR	0.09	0.11	0.02	0.02	0.14	0.30	0.40
Markers	0.22	0.33			0.01	0.19	0.20
**Achromatic**							
PhNR	0.16	0.18	0.02	0.01	0.15	–	–
Markers	0.31	0.45			0.01	–	–

For the MARS models using both the chromatic and achromatic stimulus, automated tuning removed the b-wave amplitude and it was not used as a feature, leaving 4 terms.

**Figure 2:**
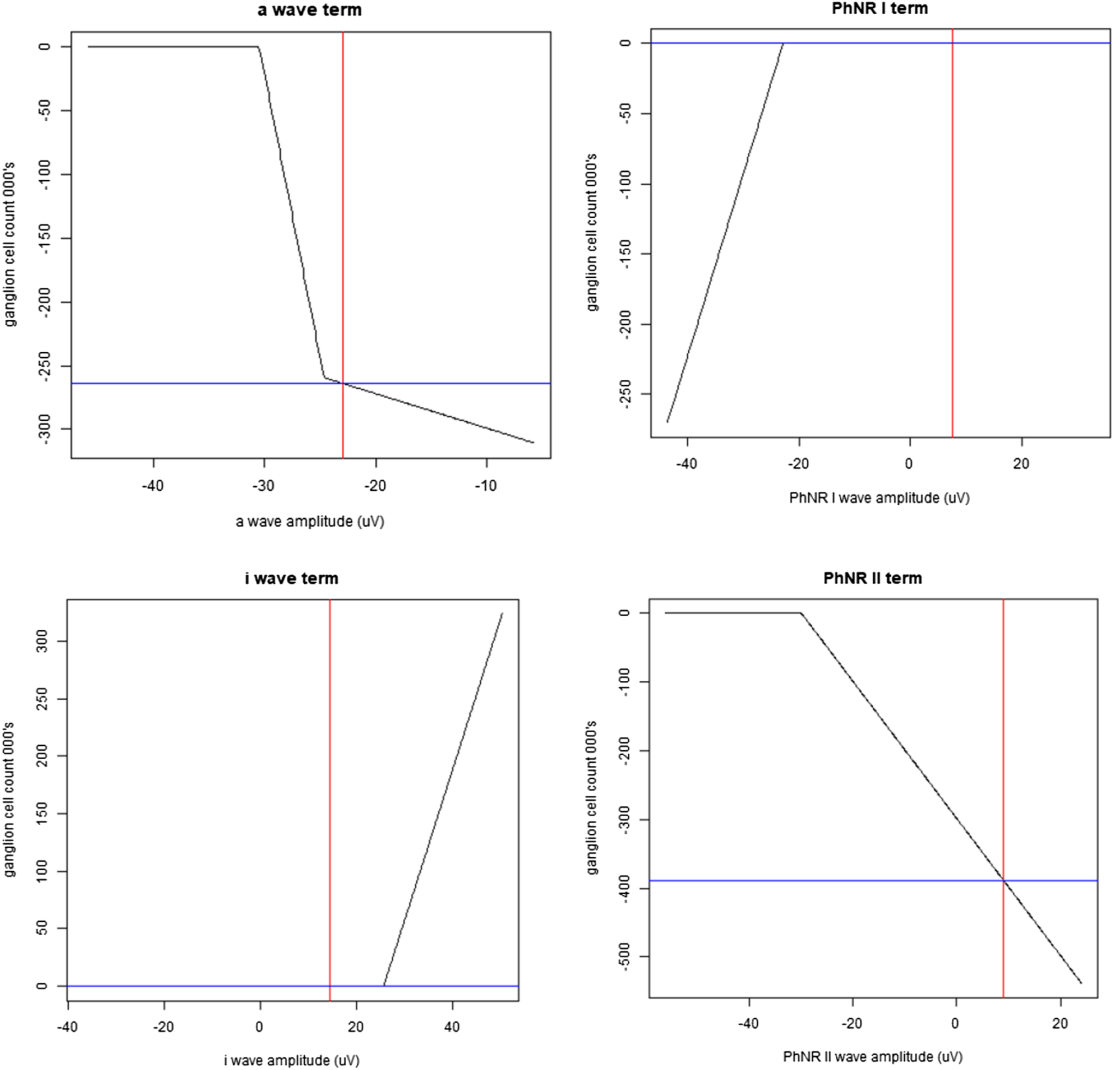
MARS model—achromatic stimulus. The panels represent the hinge functions and the influence of each term on the predicted estimate of retinal ganglion cells (eRGC). The red line shows amplitude of the various waves from an example eye. The effect on the eGRC for each term is shown on the vertical axis in each panel. The final eRGC is the intercept (940,162 retinal ganglion cells) plus the effect of each term.

The achromatic MARS marker model is illustrated in Fig. 2 with an example prediction. This model has an intercept of 940,162 RGCs and the additional effect of each term is read from the y axis. In this example, the PhNR2 and i-wave have no effect. The a-wave and PhNR2 terms contribute to give an overall estimate of 287,902 RGCs, being similar to the estimate of 322,678 RGCs for this example determined from the visual field and OCT data.

## Discussion

This study showed that using additional information from the ERG—the a-, b-, and i-wave, as well as the troughs on either side of the i-wave (PhNR1 and PhNR2)—improved the prediction of the eRGC measure compared to using the PhNR alone. Further improvements were achieved by replacing a linear model with the quasi-linear MARS model, whilst the use of achromatic stimuli resulted in non-significant improvements compared to the use of chromatic stimuli. These findings demonstrate how there is a wealth of information present in the ERG for characterizing RGC function beyond the PhNR measure alone.

The PhNR amplitude has previously been shown to correlate with glaucoma severity^17,21,23^, but this is the first time to our knowledge that multiple parameters—namely, the a-, b-, and i-waves, and the PhNR1 and PhNR2 measures—from the ERG have been used in combination to predict glaucoma severity. This study showed that the additional parameters led to significantly better performance than using the conventional PhNR measure alone, which may be attributed to the ability of such a model to allow for overall eye gain and to also to allow for the effect of the i-wave contamination of the measurement of the process driving the PhNR itself. It also allows for the inclusion of the process driving the i-wave which might itself be affected in glaucoma. We also found that the MARS models performed better than the linear models, while still allowing for a high level of intuition. As can be seen from Fig. 2 for the responses from an achromatic stimulus, the a-wave amplitude has an impact at smaller amplitudes, perhaps correcting for overall eye gain. The influence of the PhNR2 term saturates at larger (negative) amplitudes whereas the PhNR1 term only starts having an effect at large (negative) amplitudes. The i-wave only had an influence on the model when its amplitude was high. Piecewise linear functions like these are discontinuous in the first derivative and although this is unlikely to be the optimum representation of the contribution of the input features, it can be an improvement on a simple linear model in that it allows saturation. These findings highlight how non-linear models could make better use of the multiple parameters from the ERG in characterizing RGC function than described by linear models.

In this study, we did not find any significant difference between chromatic and achromatic stimuli in their prediction performance of glaucoma severity. We tested the hypothesis that the response with a white-on-white stimulus although smaller, might have better discriminatory ability for the assessment of glaucoma severity—especially with the ability to combine the other parameters in a linear and non-linear way. Although the achromatic stimulus was not worse, it did not provide any significant advantage.

Whilst this study sought to examine whether alternative approaches with the ERG could improve the prediction of glaucoma disease severity (represented by the extent of RGC loss), we did not expect a perfect relationship since the ERG may capture RGC dysfunction that precedes its loss. However, this study provides us with an opportunity to examine whether we can better fully utilize the information available from the ERG response (using more parameters and more complex modelling) and whether different methods for eliciting the response (achromatic stimuli) would be useful for capturing RGC function. Indeed, we observed that there was benefit in considering more information derived from the ERG than the PhNR alone, and that maximum benefit from the additional parameters requires more complex modelling using the MARS approach. These findings highlight how future studies seeking to explore the utility of the ERG for tackling diagnostic dilemmas in the clinical management of glaucoma, such as of whether an eye needs treatment, what the target intraocular pressure should be, and the likelihood of disease progression, would benefit from considering additional parameters present in the ERG that are exploited through the MARS approach.

A potential limitation of this study was the use of the eRGC parameter, which previous studies have cautioned may not provide estimates of the actual number of RGCs present^35,36^. Nonetheless, these studies acknowledged that this issue of estimating the number of RGCs present differed from the clinical value of combining measures of structure and function in characterizing glaucoma severity. Indeed, we used the eRGC counts parameter in this study as a means of characterizing glaucoma severity, which we consider a strength of this study as previous studies have shown that this parameter outperformed clinical measures of structure and function used in isolation for this purpose^37–40^. We showed in this study that extracting additional information from the ERG could allow better understanding of RGC function. This study was limited to simple amplitude measures, and it is possible that better performance could be obtained by adding features such as latency, frequency domain characteristics, such as median frequency or even features from time–frequency analysis such as wavelet coefficients.

Finally, another potential limitation of this study was its sample size and the inclusion of both eyes from each participant. The Intra-cluster correlation coefficient (ICC) calculated by the method of Lohr^41^ using the psych package in R^42^ yielded an ICC for the eRGC using the side as the clustering parameter of only 0.011 indicating near independence. Our study had 79% power to detect a difference of 0.10 in the R^2^ if only one eye per participant was included, based on a one-sided alpha of 0.05. Based on the effective sample size after accounting for the between-eye correlations for participants where both eyes were included, this study would have 96% power to detect this same effect size, when using the appropriate statistical methodology to account for between eye correlations (performed using the bootstrap method in this study, resampling at the individual level). The study was thus adequately powered to detect the differences in predictive performance across the different ERG models, as evident from the significant improvements detected when using the MARS model and full set of ERG features.

In conclusion, this study shows that there is significant benefit in considering more information derived from the ERG than the PhNR alone. Maximum benefit from the additional parameters requires more complex modelling, and we showed a high predictive performance for glaucoma severity using the MARS approach with the full set of ERG amplitude features. These findings highlight key analytical approaches to incorporate for future studies evaluating the utility of the photopic ERG in the clinical management of glaucoma.
